# Increased SARS-CoV-2 Testing Capacity with Pooled Saliva Samples

**DOI:** 10.3201/eid2704.204200

**Published:** 2021-04

**Authors:** Anne E. Watkins, Eli P. Fenichel, Daniel M. Weinberger, Chantal B.F. Vogels, Doug E. Brackney, Arnau Casanovas-Massana, Melissa Campbell, John Fournier, Santos Bermejo, Rupak Datta, Charles S. Dela Cruz, Shelli F. Farhadian, Akiko Iwasaki, Albert I. Ko, Nathan D. Grubaugh, Anne L. Wyllie, the Yale IMPACT Research Team

**Affiliations:** Yale School of Public Health, New Haven, Connecticut, USA (A.E. Watkins, D.M. Weinberger, C.B.F. Vogels, A. Casanovas-Massana, A.I. Ko, N.D. Grubaugh, A.L. Wyllie);; Yale School of the Environment, New Haven (E.P. Fenichel);; Connecticut Agricultural Experiment Station, New Haven (D.E. Brackney);; Yale University School of Medicine, New Haven (M. Campbell, J. Fournier, S. Bermejo, R. Datta, C.S. Dela Cruz, S.F. Farhadian, A. Iwasaki, A.I. Ko);; Howard Hughes Medical Institute, New Haven (A. Iwasaki)

**Keywords:** 2019 novel coronavirus disease, coronavirus disease, COVID-19, severe acute respiratory syndrome coronavirus 2, SARS-CoV-2, viruses, respiratory infections, zoonoses, saliva, diagnostics, screening

## Abstract

We analyzed feasibility of pooling saliva samples for severe acute respiratory syndrome coronavirus 2 testing and found that sensitivity decreased according to pool size: 5 samples/pool, 7.4% reduction; 10 samples/pool, 11.1%; and 20 samples/pool, 14.8%. When virus prevalence is >2.6%, pools of 5 require fewer tests; when <0.6%, pools of 20 support screening strategies**.**

Limited laboratory capacity in the United States has hindered access to testing for severe acute respiratory syndrome coronavirus 2 (SARS-CoV-2) and has delayed results. To control outbreaks of coronavirus disease (COVID-19), testing capacity must be increased and maintained for the foreseeable future. One resource-saving, capacity-increasing approach is pooling samples, thereby testing multiple persons simultaneously. A negative result for the pool indicates that all samples were below the limit of detection, and a positive result for the pool requires individual retesting of all samples. Pooled testing has been widely proposed as a way to expand capacity for large-scale screening (*1*,*2*; C.M. Verdun, unpub data, https://doi.org/10.1101/2020.04.30.20085290), a proactive strategy for early pathogen detection, primarily for persons who are not yet symptomatic.

Saliva is being used as a noninvasive source for SARS-CoV-2 testing (*3**,**4*) yet can be more difficult to process than traditional swab-based samples (*5*). Given limited empirical evidence to properly inform projections of feasibility and cost-effectiveness of pooling, we explored the potential of pooling saliva to increase SARS-CoV-2 testing capacity**.**

## The Study

**Figure 1:**
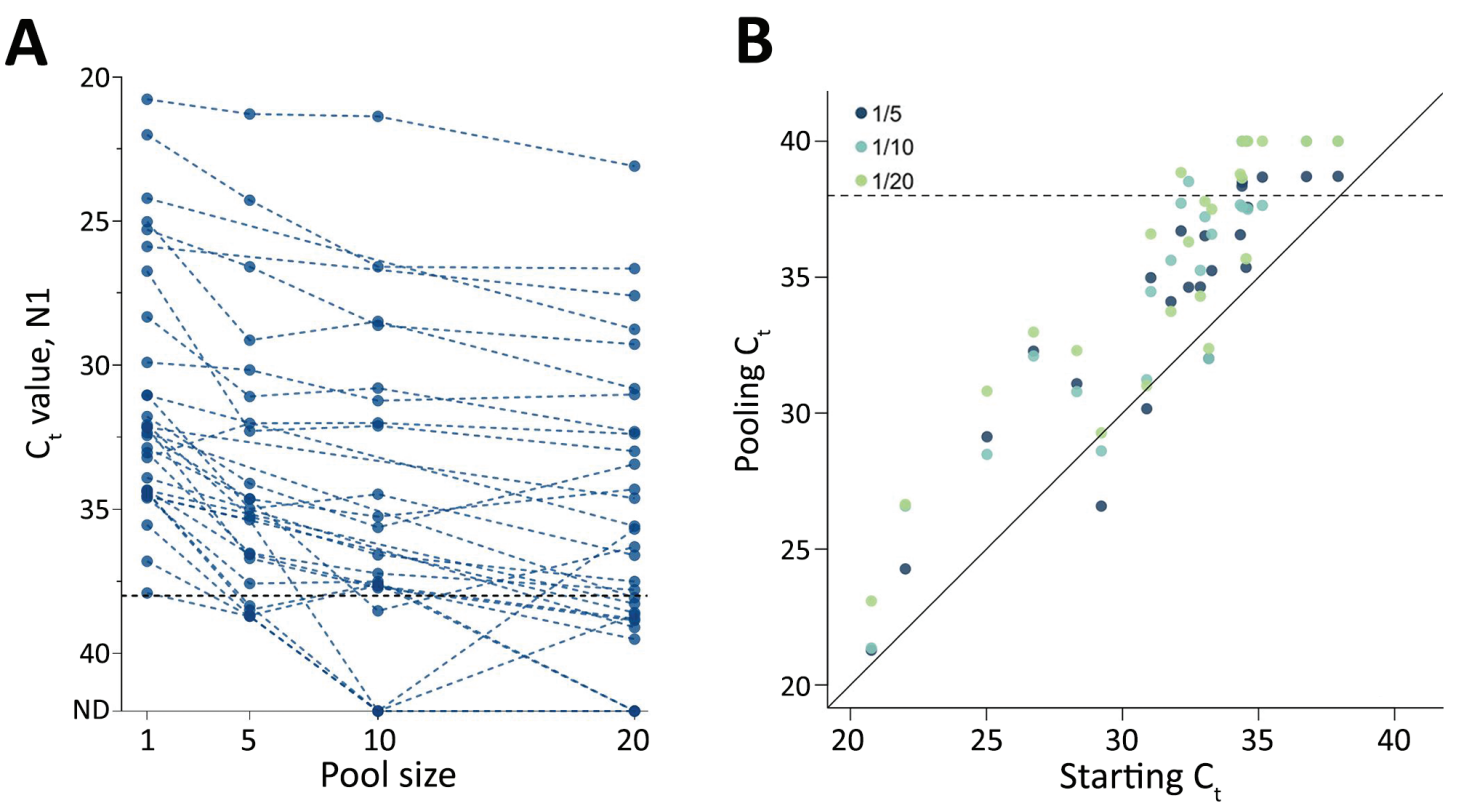
Effect of pooling on detection of severe acute respiratory syndrome coronavirus 2, by pool size and between samples tested. A) As the pool size increased, so did the C_t_ value (dotted lines connect pools comprising the same positive sample). C_t_ for positivity is set to 38. Samples falling on the x-axis indicated samples from which signal was not detected by reverse transcription quantitative PCR. B) As the pool size increased, so did the C_t_. We equated this change by using linear regression (pool of 5 samples, dark blue, +2.2 C_t_, 95% CI 1.4–3.0 C_t_; pool of 10, light blue, +3.1 C_t_, 95% CI 2.3–4.0 C_t_; pool of 20, green, +3.6, 95% CI 2.7–4.4 C_t_). Dashed lines indicate C_t_ 38 (cutoff for sample positivity). 1/5, pool of 5; 1/10, pool of 10; 1/20, pool of 20. C_t_, cycle threshold.

Using saliva collected from COVID-19 inpatients and at-risk healthcare workers (*5*), we combined 1 SARS-CoV-2–positive sample (<38 PCR cycle threshold [C_t_]) with SARS-CoV-2–negative saliva (Appendix) before RNA extraction in total pool sizes of 5 samples/pool (n = 23 pools), 10 (n = 23), and 20 (n = 31). As pool size increased, detection sensitivity decreased independent of starting viral load (pool of 5, +2.2 cycle threshold [C_t_], 95% CI 1.4–3.0 C_t_; 10, +3.1 C_t_, 95% CI 2.3–4.0 C_t_; 20, +3.6 C_t_, 95% CI 2.7–4.4 C_t_) (Figure 1; Appendix).

By applying the regression coefficients (C_t_ increase) to the C_t_ values from all SARS-CoV-2–positive saliva samples detected during our studies (*6*), we estimate that pool sizes will lead to detection sensitivities of 92.59% (95% CI 88.89%–95.56%) for pools of 5 samples, 88.89% (95% CI 80.00%–91.85%) for pools of 10, and 85.19% (95% CI 75.56%–91.11%) for pools of 20, relative to sensitivity of unpooled samples (Appendix Figure 1). This loss in sensitivity could be minimized through protocol modifications: increasing the volume of pooled samples tested (400 μL, n = 20 pools of each size; Appendix Figure 2) and decreasing the elution volume.

**Figure 2:**
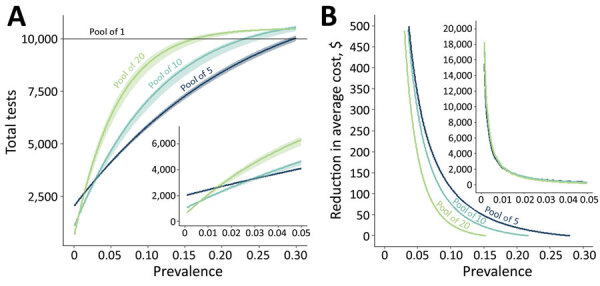
The resource-saving benefit of sample pooling for severe acute respiratory syndrome coronavirus 2 (SARS-CoV-2) testing, based on size of the pool and expected prevalence of SARS-CoV-2 within the population. A) We modeled the number of tests required to test 10,000 persons (results qualitatively scale with population) when pools contain 5, 10, or 20 samples (and individually retesting samples within positive pools) compared with testing samples individually (pool = 1 sample). As prevalence increases, so does the number of pools positive for SARS-CoV-2, thereby increasing the required number of confirmatory tests of individual samples. Therefore, over a prevalence of 2.6%, pooled samples of 5 result in fewer overall tests required than do larger pool sizes. B) At lower prevalence rates, such as when outbreaks have been controlled but ongoing screening is required, pools of 10 or 20 samples yield substantial cost savings for the same expected level of positive detections, after accounting for sensitivity differences. Values are shown in US$. Insets show the region with <5% prevalence.

On the basis of the calculated relative sensitivity loss resulting from pooling, we modeled the number of tests required (total of pooled and individual samples from positive pools tested) for a population of 10,000 with increasing SARS-CoV-2 prevalence (Figure 2, panel A). We estimate that for populations with prevalence <0.6%, pools of 20 require the fewest tests. However, for populations with prevalence >2.6%, our analyses suggest that pooling of 5 samples leads to the fewest tests. For populations with prevalence >28.1%, testing individual samples is more efficient than testing pools of any size. Thus, we suggest using an adaptive pooling strategy that accounts for SARS-CoV-2 prevalence for the population tested: as virus prevalence decreases, pool size can be increased, but as prevalence rises, pool size should be decreased.

Because sensitivity varies by pooling design (Figure 1), a different number of positive results will be detected for a given population with a given SARS-CoV-2 prevalence. As virus prevalence decreases, we estimate that cost savings of pooled testing will increase (Figure 2, panel B). For example, if SARS-CoV-2 prevalence for a 10,000-person population was 0.5%, then pooling by 20 would require only 1,318 tests, including retesting of all persons from test-positive pools. If tests cost US$30 each, the savings would be $260,453 relative to individual testing while still identifying ≈43 of 50 infected persons. The savings will vary on a scale relative to test prices. Ultimately, the net benefits of pooled testing can continue to increase even as virus prevalence decreases with increased pool sizes, which is essential for ongoing screening.

## Conclusions

The cost of SARS-CoV-2 testing can be prohibitive when positive samples are rarely found, presenting a major barrier to prolonged screening strategies. Pooling of samples can help overcome this barrier. Our model demonstrates that as local outbreaks fluctuate, adapting pool sizes will have resource-savings benefits.

The benefits of pooled testing will always be accompanied by decreased detection sensitivity. However, the lower overall number of tests required and the lower associated costs expands testing capacity, permitting more frequent testing, and testing persons more often mitigates the loss of sensitivity (*7*). By enabling broader testing, pooling has the potential to identify more infected persons than more limited (or no) individual testing. Infected persons can then be isolated from the population, thus reducing the probability of contact between a susceptible and an infectious person, ultimately reducing transmission. Given our findings, we urge the US Food and Drug Administration to develop new guidelines for pooled-testing approaches. Although the first Emergency Use Authorization for SARS-CoV-2 pooled testing (<4 swab samples in 1 test) (*8*) will be most useful in high-prevalence settings, the ≈12%–15% losses in sensitivity when pooling 10–20 samples would probably not pass current authorization criteria (>95% sensitivity).

Going forward, screening strategies need to be reviewed separately from traditional diagnostic testing, taking into consideration the repeated testing of individuals performed during screening. For strategies considering twice-weekly sampling, such as in the reopening plans for many US colleges, even if larger pools have lower sensitivity per test, the probability of 2 repeated false-negative results for any person will often be less than the probability of a false-negative result for a single test from a small pool. For example, a small pool (or individual test) may have the probability of a false-negative result of 2% but cost may limit testing to once per week. Conversely, the lower per-person cost of a large pool with a per-test probability of a false-negative result of 14% is more likely to allow for testing twice per week. Therefore, persons tested twice in larger pools have a per-week false-negative probability of only 1.96%. In the context of prolonged community screening, sensitivity should be thought of as per unit time, and the testing regimen should be taken into account.

Our estimates are conservative; the number of tests required is most likely lower than predicted, especially if behavioral or geographic information can be used to stratify the population so that the adaptive pooling strategy can be applied differentially to different sampled subpopulations. However, this approach needs to be balanced with feasibility in the laboratory because pooled testing adds additional steps and complexity to the system, all of which must be reliably implemented. Furthermore, pooled approaches could incorporate retesting individual samples from pools generating any SARS-CoV-2–specific signal in quantitative reverse transcription PCR regardless of C_t_ (in place of those pools with the <38 C_t_ cutoff applied here) (*9*). Although pooling has traditionally focused on extracted nucleic acid before quantitative reverse transcription PCR (*10**–**12*), because of the expense of RNA extraction and a comparable effect on detection sensitivity (Appendix), we recommend pooling before RNA extraction. Validation of our work in additional settings and on a larger scale will help better inform our models.

The cost-savings benefits of adaptive pooling saliva for community screening for SARS-CoV-2 provides a mechanism to maintain testing as virus spread is brought under control and to avoid resurgence. Even if prevalence is very low, it is probably desirable to increase pool sizes before stopping testing altogether. Together with the ease of saliva collection, pooling samples should be considered as an effective testing strategy for expanding the breadth of testing and continued screening during the ongoing COVID-19 pandemic.

Appendix Additional methods and results for study of pooling saliva to increase SARS-Cov-2 testing capacity.
