# Supplementary material for: Increased SARS-CoV-2 Testing Capacity with Pooled Saliva Samples
Source: Emerg Infect Dis. 2021 Apr;27(4):1184–7. doi: 10.3201/eid2704.204200 (PMC8007323; doi:10.3201/eid2704.204200)
Supplement: Appendix — Additional methods and results for study of pooling saliva to increase SARS-Cov-2 testing capacity. [file 20-4200-Techapp-s1.pdf]

Article DOI: https://doi.org/10.3201/eid2704.204200

Increasing SARS-CoV-2 Testing Capacity with Pooled Saliva Samples

Appendix

Methods

Sample pooling

Saliva was collected as a part of the Yale IMPACT Biorepository (*1*) from COVID-19 inpatients and healthcare workers at the Yale-New Haven Hospital (Yale Human Research Protection Program Institutional Review Boards FWA00002571, Protocol ID. 2000027690) (*1*). RNA was extracted and tested by RT-qPCR for SARS-CoV-2 RNA (N1) (*2*).

Saliva samples were combined into pools of 5 (n=23), 10 (n=23), and 20 (n=31). Each pool contained equal amounts of one SARS-CoV-2 positive sample (as determined by RT-qPCR; 31 positive samples total) and the respective number of individual SARS-CoV-2 negative samples required to complete the target pool size. RNA extraction from pooled samples and RT-qPCR for SARS-CoV-2 detection were performed according to the biorepository’s standard operating procedures (*1*–*3*) with either 300 µl (equating to 60 µl, 30 µl, and 15 µl of the original sample) or 400 µl (equating to 80 µl, 40 µl, and 20 µl of the original sample; n=20 for each of 5, 10 and 20 pools) total extraction input volume with RNA eluted into a total volume of 75 µl. Later, RNA extracted from saliva (n=10) was tested individually or together in pool sizes of 5 or 10 and tested in RT-qPCR for SARS-CoV-2 detection (*2*). The cut-off for all RT-qPCR assays in this study was set at a cycle threshold (Ct) of 38 (*2*).

Statistical analyses

Sensitivity analyses

We fit a linear regression to the experimental pooling data to model the change in Ct values of positive samples following pooling. Let ‘ΔCt’ be the change in Ct value of pooled samples and let ‘ratio’ be the categorical ratio of pool size (i.e. 1/5, 1/10, 1/20). Analyses were done separately by input volume in order to determine the effect of pool size under both 300 µl

and 400 µl extraction conditions. This equation was used, separately, for both pre-extraction saliva and post-extraction RNA pooling. Ratio in this model can be interchanged with "condition" for the model of the 1/20 PBS and water dilution data.

We found that the change in Ct value post-pooling was independent of the Ct value of the undiluted sample (Pearson's, r=-0.004; 95% CI: -0.240, 0.233), thus it was not included in the model. Confidence intervals were generated by simulating from the covariance matrix of the parameters from the fitted model using the mvrnorm function in the R package "MASS" (*4*), and quantile functions.

Modeling the resource-saving benefit of sample pooling for SARS-CoV-2 testing

The problem of pooling can be approached modularly. We model pooling based on the expected prevalence in a test population of known size at a given time. By approaching the problem this way, we abstract from the problem of estimating prevalence in the sampled population at a given time. Nevertheless, our approach can be plugged into broader population level models with epidemiological dynamics.

If samples are independent of each other, pulled from the same well-mixed population (identically distributed), and that anyone in a test-positive pool needs to be re-tested individually, then binomial sampling theory provides the tool to compute the number of tests needed, which has been used for over half a century (*5*,*6*). The number of positive groups is $P = [1 - (1 - \sigma(g)m)^g](N/g)$, given a total test population of size N that is divided into groups of size g yield (N/g) groups, with a prevalence of infection in the sampled population equal to m and a test sensitivity $\sigma(g)$, where sensitivity can be a function of group size. The total number of tests need is $T = (N/g) + Pg$. The R script to implement these calculations are available at https://github.com/efenichel/pooled-saliva-testing.

To calculate the total number of tests and the number of test positive groups, we assume the expected prevalence is computed with error or that any error is orthogonal to the sampling error associated with the estimates of sensitivity. Therefore, to propagate the uncertainty associated with sensitivity sampling error, we make the calculations for the number of positive groups and total tests using a single predefined, conservative cut-off value. This mimics the existence of an established protocol. Variation in the cycle thresholds used would increase the sampling uncertainty for sensitivity, and would expect the point estimate of the sensitivity,

conditional on pool size, to be a convex combination of the estimates using individual cycle thresholds.

In practice, those coordinating testing need to consider what constitutes a single, well-mixed sampled population. For example, demographic or socio-economic information may be used to group samples into distinct subpopulations prior to pooling and testing. This would be called stratifying the population. If these subpopulations have different expected prevalences, then different sized pools may be optimal for the different subpopulations. However, stratification requires population specific data that is invariant to the test itself or stronger assumptions. The possibility of embedding an adaptive pooling approach into a model of a system that brings population level data to bear is a strength of the approach.

Another consideration that the model does not directly address is selection into the sampled population. If there is selection, then the well-mixed assumption is violated. There are two reasons to be concerned about this in practice. First, if people who are more likely to test positive are also more likely to get tested when there is a binding test capacity constraint, then as the constraint is relaxed with pooling, the expected prevalence in the population is likely to fall. This is a reason why stratifying the sample based on observable features, e.g., self-assessed probability of infection prior to pooling might be important. Conversely, consider a segment of the population that tries to avoid testing and engages in high risk behaviors (i.e., people who believe COVID-19 is a hoax). These people select out of testing. If it is easier to include these people in a testing regime with greater capacity due to pooling, then expected prevalence may actually rise. This can also be addressed with stratification of the population.

Further statistical analyses were conducted in GraphPad Prism 8.0.0 as described in the text and figure legends.

Results

When pooling saliva samples, the effect on the sensitivity of detection was independent of the Ct value of the undiluted sample (Pearson's, r=-0.004; 95% CI: -0.240, 0.233), i.e. the sensitivity loss in a sample with a higher Ct value (lower viral load) was not more than that of a sample with a lower Ct value (higher viral load).

We also evaluated the effect of pooling post-RNA extraction and pooled RNA templates extracted from undiluted saliva samples by 5 and by 10 (n=10). While we observed a similar decrease in sensitivity (pool: of 5, +2.2 Ct, 95% CI: 1.7-2.6; pool of 10, +3.1 Ct, 95% CI: 2.6-3.6) as to when pooled prior to RNA extraction, the degree to which each sample varied was less with less overall variation as compared to pre-extraction pooling (F test, pools: of 5, p = 0.061; pools of 10, p = 0.009, Appendix Figure 3).

References

1. Wyllie AL, Fournier J, Casanovas-Massana A, Campbell M, Tokuyama M, Vijayakumar P, et al. Saliva or nasopharyngeal swab specimens for detection of SARS-CoV-2. N Engl J Med. 2020;383:1283–6. PubMed https://doi.org/10.1056/NEJMc2016359

2. Vogels CBF, Brito AF, Wyllie AL, Fauver JR, Ott IM, Kalinich CC, et al. Analytical sensitivity and efficiency comparisons of SARS-CoV-2 RT-qPCR primer-probe sets. [Internet]. Nat Microbiol. 2020;5:1299–305. PubMed https://doi.org/10.1038/s41564-020-0761-6

3. Ott I, Vogels C, Grubaugh N, Wyllie A. Saliva collection and RNA extraction for SARS-CoV-2 detection v1 [cited 2020 Dec 12]. https://doi.org/10.17504/protocols.io.bg3pjymn

4. Ripley B, Venables B, Bates DM, Hornik K, Gebhardt A, Firth D, et al. Package "MASS." [cited 2021 Jan 28]. https://cran.r-project.org/web/packages/MASS/MASS.pdf

5. Dorfman R. The detection of defective members of large populations. Ann Math Statist. 1943;14:436–40. https://doi.org/10.1214/aoms/1177731363

6. Gollier C, Gossner O. Group testing against Covid-19 [cited 2021 Jan 28]. Covid Economics. 2020;1:32–42.

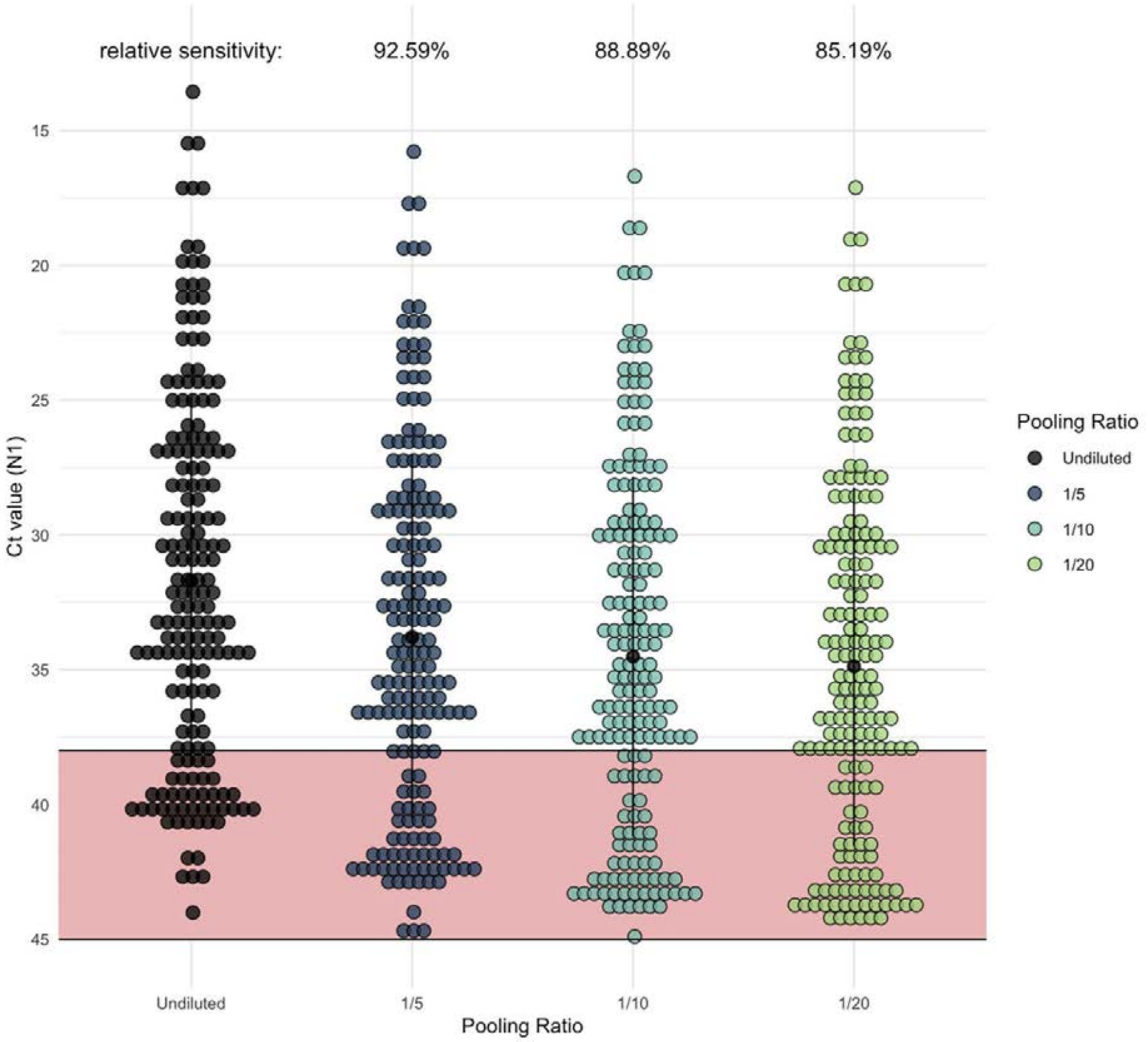

Appendix Figure 1. As pool size increases, more samples would be classified as negative in comparison to samples tested individually (unpooled). Each dot represents one of the 180 Yale IMPACT saliva samples which generated signal when tested by RT-qPCR for SARS-CoV-2 N1. Of these, 135 fell below the cycle threshold (Ct) of 38 (solid line) and were classified as positive for virus. The regression coefficient (representing expected Ct increase) for each of the pool sizes was added to the Ct value generated from the undiluted sample (shown in black) to determine the relative level of sensitivity for each pool size. The area shaded in red indicates the Ct range in which N1-signal is considered to be below the limit of detection.

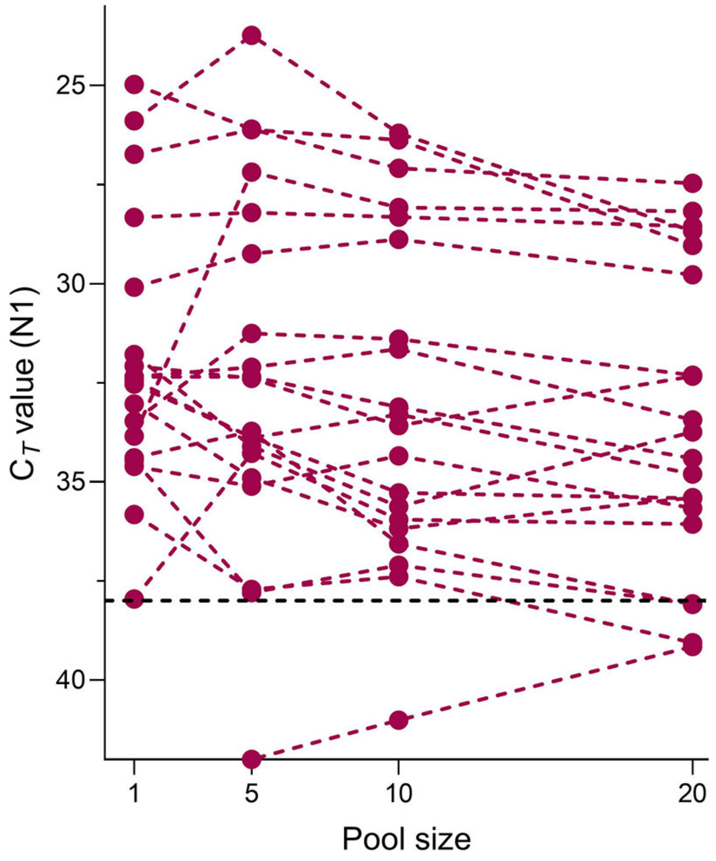

Appendix Figure 2. Cycle threshold (Ct) values of saliva samples (n=20) tested individually (pool size = 1) at a total volume of 300 µL, or when diluted with an increasing number of negative samples (total pool sizes of 5, 10 and 20) and a total extraction volume of 400 µL. When extracting from 400 µL volumes of pooled samples, we observed improved detection (pool of 5, -0.1 Ct, 95% CI -1.2, 1.1; pool of 10, 0.3 Ct, 95% CI -0.8, 1.5; pool of 20, 1.1 Ct, 95% CI -0.1, 2.2; linear regression). Dotted lines connect pools comprised of the same positive sample. Ct threshold for positivity is set to 38. Samples falling below the x-axis indicated samples from which signal was not detected in RT-qPCR.

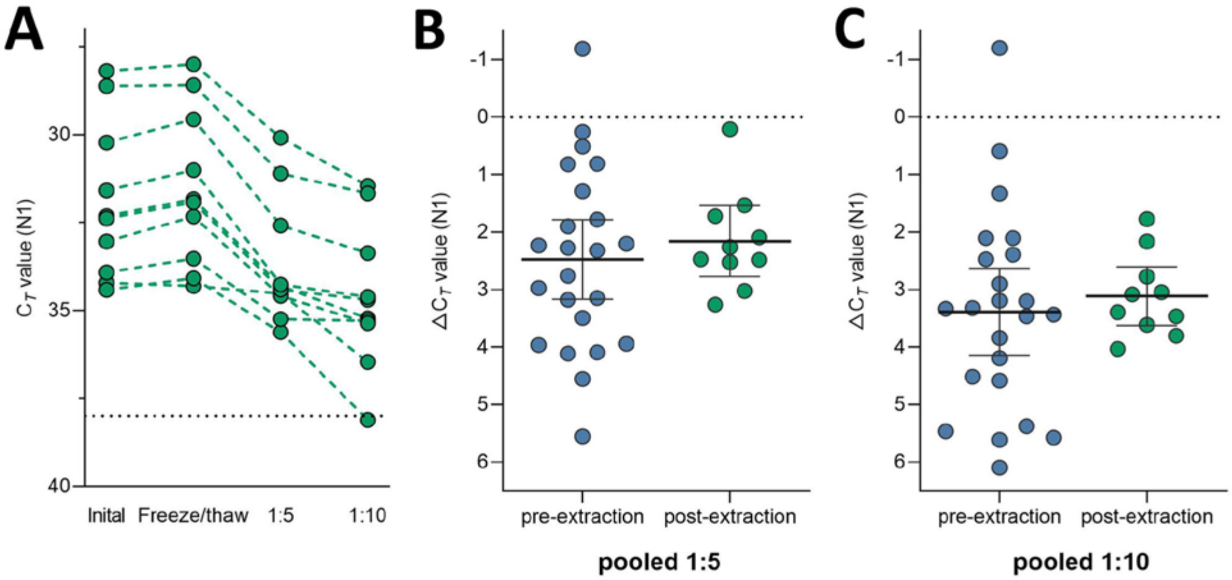

Appendix Figure 3. Less variation in cycle threshold (Ct) values when pooling RNA templates. (A) Ct values of SARS-CoV-2 positive RNA (n=10) extracted from saliva samples when tested individually (pool size = 1) on day of sample collection (initial) and following storage of RNA at -80°C (freeze/thaw), or when diluted with 4 or 9 SARS-CoV-2 negative RNA templates (total pool sizes of 5 and 10). Dotted lines connect pools comprised of the same positive sample. While the median change in Ct value was comparable whether pooling samples or RNA templates by (B) 5 (Mann-Whitney, $p = 0.499$) or (C) 10 (Mann-Whitney, $p = 0.556$), pooling of samples resulted in more varied Ct changes (F test, $p = 0.061$ and $p = 0.009$, respectively).
